# Ergot of Rye in Leucorrhœa

**Published:** 1831

**Authors:** 


ERGOT OF RYE IN LEUCORRHŒA.

IN the same Italian Journal, and of the same date, we observe a long paper from Dr. Bazzoni, on the use of ergot of rye in leucorrhœa. He details a considerable number of cases, in most of which the medicine appears to have produced decided effects. A single case, the first on the list. A female, aged 38 years, of feeble constitution, but regular in her menstrual periods, experienced some severe mental affliction at the expected time of menstruation, and was seized with severe abdominal pains in their stead, accompanied by head-ache, fever, &c. which were removed by bleeding and diluents. At the succeeding period, the menses were replaced by a fluor albus, preceded and accompanied by abdominal pains and distressing sickness, which continued for five or six days. The third period presented the same phenomena, except the leucorrhœal discharge continued much longer. After this the fluor albus seemed to have become habitual, attended with pains in her loins, bad digestion, and much geneneral debility. At this time Dr. Bazzoni was consulted, and prescribed 20 grains of the ergot of rye to be boiled in eight ounces of water, and taken in the course of two days. No inconvenience was felt from the medicine, and on the third day the leucorrhœa had disappeared. On the succeeding month the menses appeared, and continued regular afterwards.

---

XLVI.

